# Pulsed electromagnetic field attenuated PTSD-induced failure of conditioned fear extinction

**DOI:** 10.22038/ijbms.2019.32576.7797

**Published:** 2019-06

**Authors:** Mohammad Ali Mohammad Alizadeh, Kataneh Abrari, Taghi Lashkar Blouki, Mohammad taghi Ghorbanian, Majid Jadidi

**Affiliations:** 1 School of Biology, Damghan University, Damghan, Semnan, Iran; 2 Department of Medical Physics, School of Medicine, Semnan University of Medical Sciences, Semnan, Iran

**Keywords:** Classical conditioning Electromagnetic fields, Hippocampus, Neurogenesis, Post-traumatic stress - disorder

## Abstract

**Objective(s):**

This study aimed to determine whether exposure to pulsed electromagnetic field (PEMF) can impair behavioral failure as induced by PTSD, and also its possible effects on hippocampal neurogenesis. PEMF was used as a non-invasive therapeutic tool in psychiatry.

**Materials and Methods:**

Male rats were divided into Control-Sham exposed, Control-PEMF, PTSD-Sham exposed, and PTSD-PEMF groups. PTSD rats were conducted by the single prolonged stress procedures and then conditioned by the contextual fear conditioning apparatus. Control rats were only conditioned. Experimental rats were submitted to daily PEMF (7 mT, 30 Hz for 16 min/day, 14 days). Sham-exposed groups were submitted to the turned off PEMF apparatus. Fear extinction, sensitized fear and anxiety, cell density in the hippocampus, and proliferation and survival rate of BrdU-labeled cells were evaluated.

**Results:**

Freezing of PTSD-PEMF rats was significantly lower than PTSD-Sham exposed. In the PTSD-PEMF, center and total crossing in open field, also the percentage of open arms entry and time in the elevated plus maze, significantly increased as compared with PTSD-Sham exposed (*P*<0.001). Numbers of CA1, CA3, and DG cells in PTSD-PEMF and Control-Sham exposed groups were significantly more than PTSD-Sham exposed (*P*<0.001). There were more BrdU-positive cells in the DG of the PTSD-PEMF as compared with the PTSD-Sham exposed. Qualitative observations showed an increased number of surviving BrdU-positive cells in the PTSD-PEMF as compared with PTSD-Sham exposed.

**Conclusion:**

Using 14-day PEM attenuates the PTSD-induced failure of conditioned fear extinction and exaggerated sensitized fear, and this might be related to the neuroprotective effects of magnetic fields on the hippocampus.

## Introduction

The post-traumatic stress disorder is an anxiety disorder diagnosed after a person is exposed to a traumatic event such as a dangerous accident, life-threatening stressors, and so on. Symptoms such as flashbacks, nightmares, hyperarousal, and avoidance of reminders associated with the trauma carry on for at least one month following a traumatic event (1). PTSD causes physiological changes in the brain and body. Hence could have severe effects on the hippocampus, problems with memory (2), enhanced acquisition, and lack of extinction ability (3). 

Studies on PTSD patients show three brain areas with functional changes: prefrontal cortex, amygdala, and hippocampus; and these structures play a critical role in triggering the memory symptoms of PTSD (4). During stressful events, secretion of stress hormones was elevated. An excess of cortisol can cause neuronal damage within the hippocampus and impair the ability of hippocampus to both encode and recall memories (5), which may be a significant factor toward the development of PTSD. Repetitive stress suppresses the proliferation and survival of hippocampal granule cells (6). The hippocampus has central importance in PTSD due to its prominent role in both the neuroendocrine stress response and memory alterations (7). Many studies have demonstrated smaller hippocampal volumes in PTSD (4, 8). 

Considering the effect of stressful conditions on the physiology and structure of the brain and the importance of the hippocampus in memory in PTSD, discovering approaches to prevent the impact of stress hormones on brain structures, especially the hippocampus is essential. 

Over the past 10–20 years, noninvasive treatments such as pulsed electromagnetic field (PEMF) are increasingly being taken into consideration and widely used for many conditions and medical disciplines such as pain syndromes, sleep quality, RNA and DNA stimulation, depression, obsessive-compulsive disorders, refractory epilepsy, and so on. Pulsed electromagnetic fields relatively simple devices produce a series of strong magnetic pulses that create a weak electrical current can either increase or decrease activity in specific parts of the body. These devices use an external, non-invasive PEMF to generate short bursts of electrical current in injured tissue without producing heat or interfering with nerve or muscle function (9-11). 

It is extensively reported that electromagnetic fields positively modulate different steps of neurogenesis by affecting several different targets. Exposure to extremely low-frequency electromagnetic field significantly enhanced the hippocampal neurogenesis and differentiation of cortical neural stem cells *in vitro* (12, 13).

The existence of a causal link between electromagnetic fields-enhanced neurogenesis and increased synaptic plasticity is supported by some experimental studies (14, 15). Magnetic stimulation enhances adult hippocampal neurogenesis within a short period and makes it possible to study the role of adult hippocampal neurogenesis in a gain-of-function manner (16). However, neither the precise pattern of brain activation nor the molecular mechanisms underlying the behavioral effects of PEMF are known. 

Given that adult neurogenesis is an extremely dynamic process that is regulated by neuronal activity and environmental factors, and as stressful events negatively affect adult hippocampal neurogenesis and regulate the rate of neurogenesis in the adult brain, and also as EMF has the potential to modulate neurogenesis in the hippocampus, we established this research. One of the most consistent brain structural abnormalities in PTSD is decreased hippocampal volume. How changes in hippocampal volume in PTSD are manifested at the level of encoding and how stress affects adult hippocampal neurogenesis is not clear. Theoretically, when applied after the PTSD model, EMF might ameliorate PTSD induced behavioral deficits and can induce changes in brain activity. Hence, the present study aimed to determine whether a PEMF treatment might exert a beneficial effect on the PTSD-induced impairment of conditioned fear extinction, sensitized fear, and anxiety-like behaviors. We prompt to investigate whether PEMF effects on PTSD extinction deficit was mediated by the increase in adult neuron production and/or survival of the newly generated cells in the hippocampal regions.

## Materials and Methods

***Animals***

In this behavioral, histological and immunohistochemical study, ninety adult male Wistar rats (180–200 g) were obtained from the Razi Vaccine and Serum Research Institute (Iran). Animals were housed five rats in a cage and maintained on a 12-hr light/dark cycle. Food and water were provided *ad libitum*. Rats were handled for 30 sec/day for seven days to habituate them to the experimenters. Behavioral tasks were performed during the light phase of the cycle. All experiments were done following the National Institute of Health Guide for the Care and Use of Laboratory Animals (NIH Publication No 23–80, revised 1996). At the end of the experiments, animals were sacrificed under light ether anesthesia using a guillotine. 

**Figure 1:**
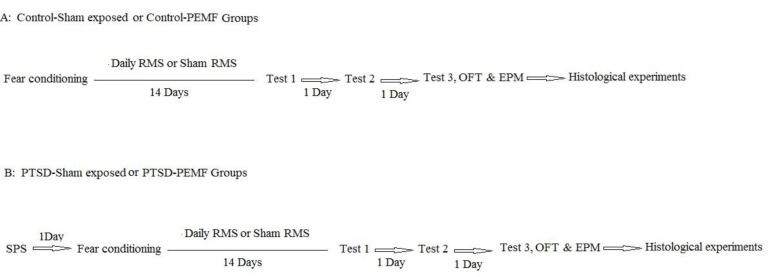
Experimental design for experimental groups. A; Control-Sham exposed and Control-PEMF groups, B; PTSD-Sham exposed and PTSD-PEMF groups

PEMF: Pulsed electromagnetic field; OF: Open field; EPM: Elevated plus maze

**Figure 2:**
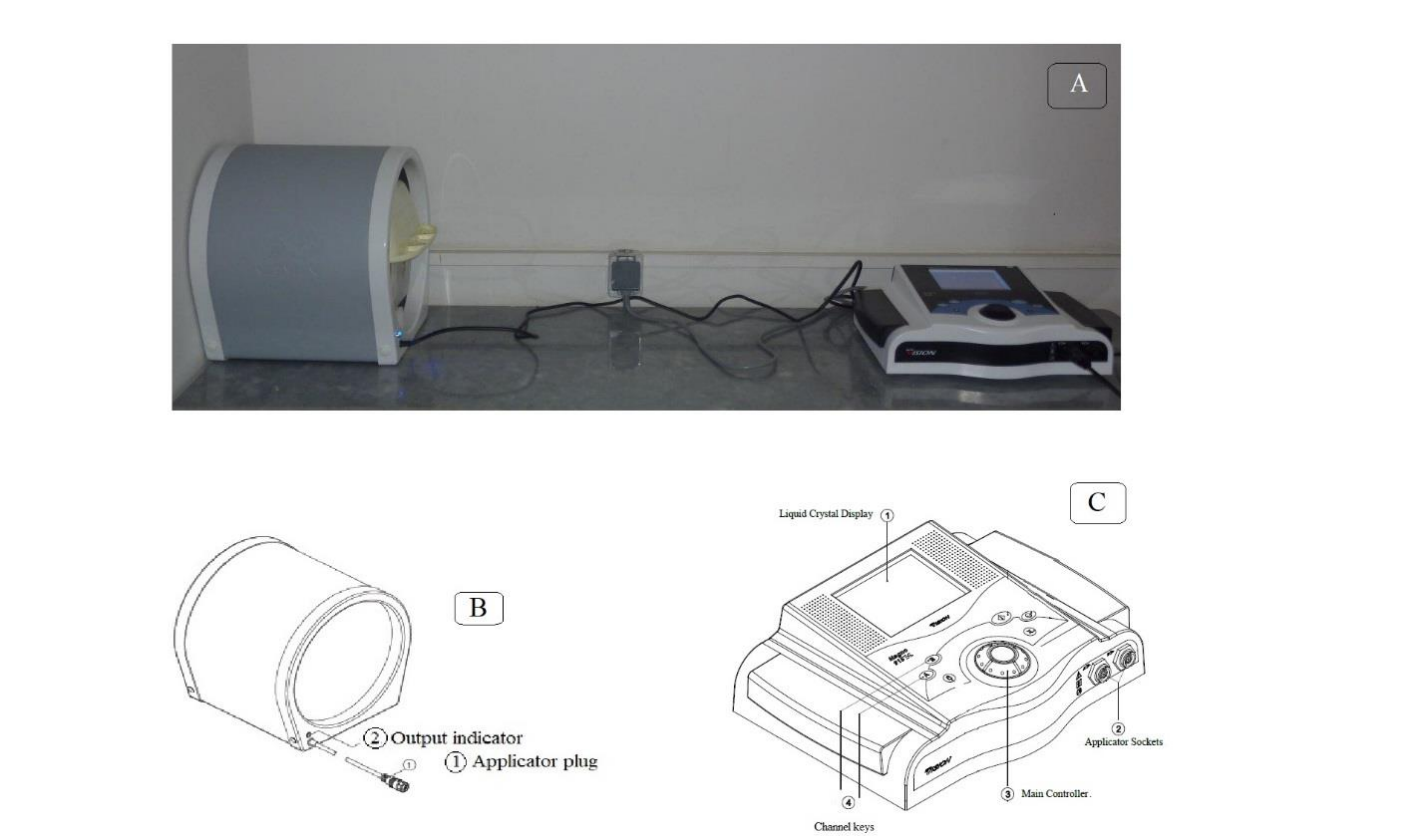
A: Electromagnet generator device (Model Magno 915X, Novin Medical Engineering Company, IRAN). B: solenoid coil C: regulator

**Figure 3:**
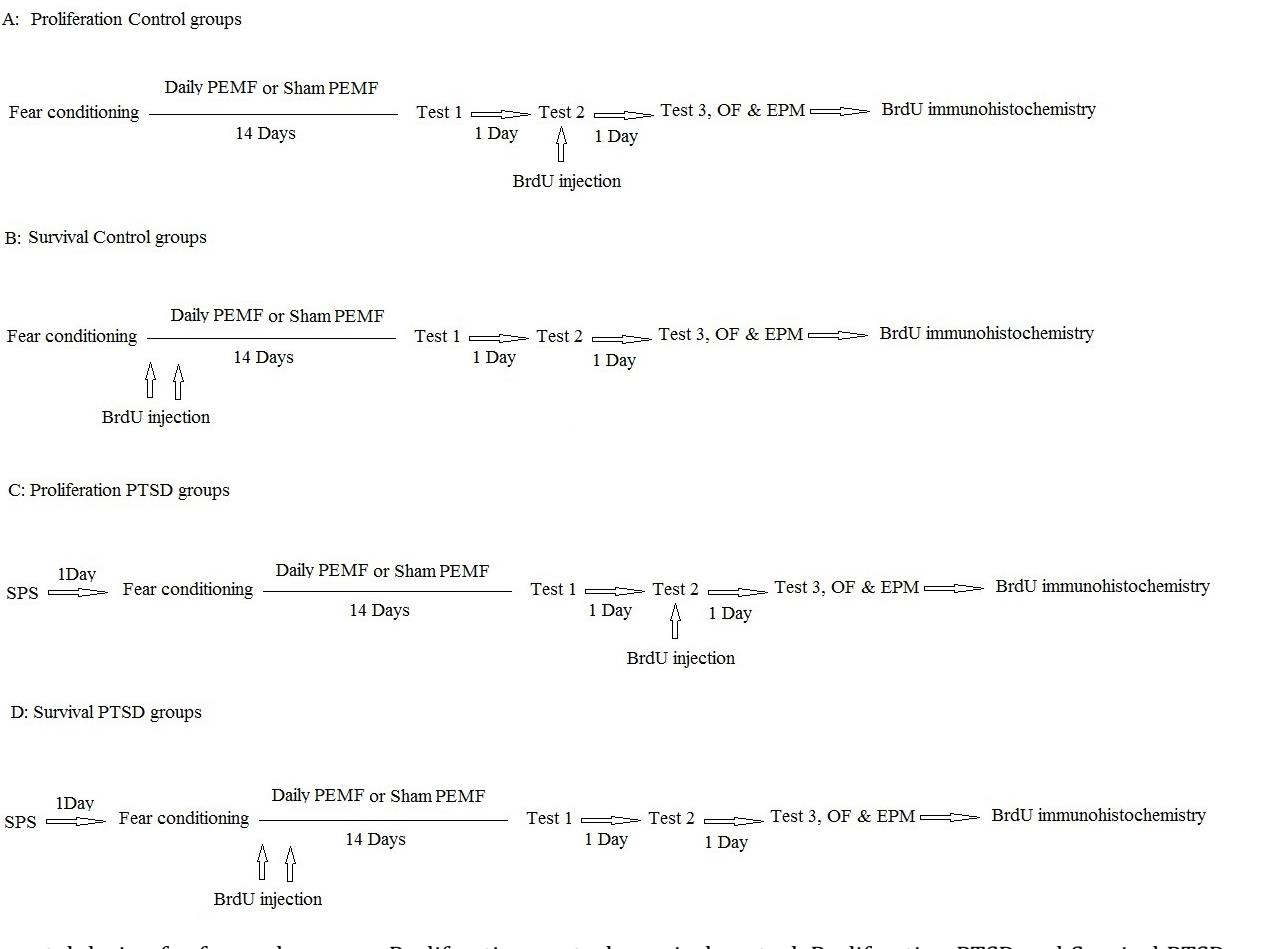
Experimental design for four sub-groups; Proliferation control, survival control, Proliferation PTSD and Survival PTSD groups. PEMF: Pulsed electromagnetic field; OF: Open field; EPM: Elevated plus maze

**Figure 4:**
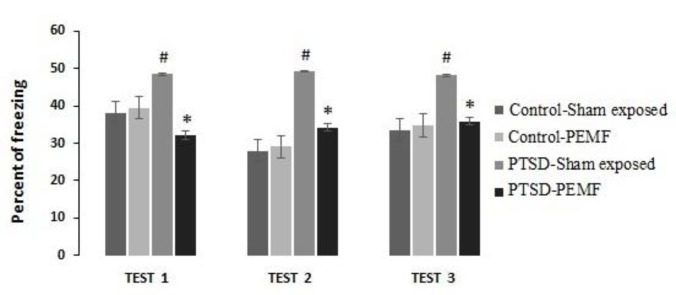
Freezing responses of different groups in conditioned fear extinction tests. Three tests over three continuous days were performed. Freezing responses assessed by the time spent in freezing (absence of all visible movement expect respiration) during 15 min test

* *P*<0.001 as compared with PTSD-Sham exposed group

# *P*<0.001 as compared with Control-Sham exposed group

**Figure 5:**
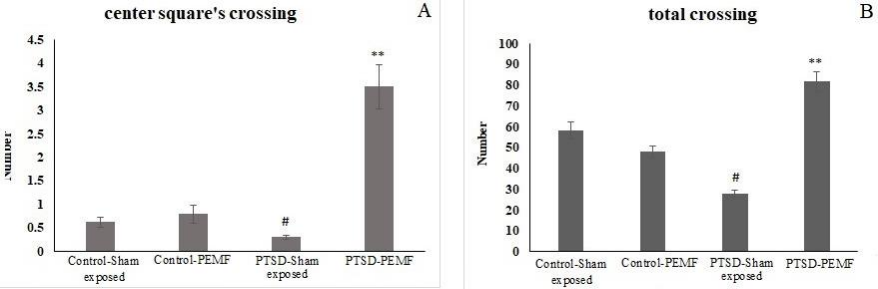
Sensitized fear test: Center square crossing (A) and total crossing (B) of different groups in the open field test were measured

** *P*<0.001 as compared with PTSD-Sham exposed group

#* P*<0.001 as compared with Control-Sham exposed group

**Figure 6:**
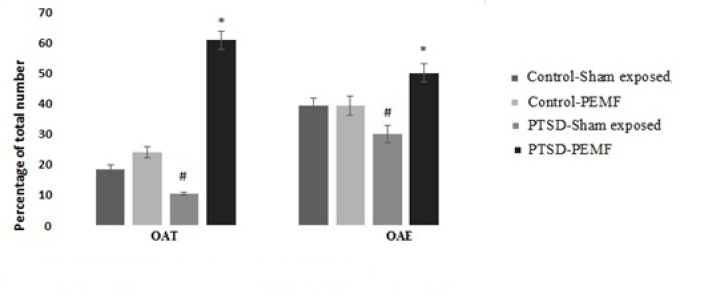
Anxiety-like behavior: The percent time spent in the open arms and also the number of entries into the open arms of the elevated plus maze were evaluated. OAT; Open arm time. OAE; Open arm entry.

* *P*<0.001 as compared with PTSD-Sham exposed group

# *P*<0.001 as compared with Control-Sham exposed group

**Figure 7:**
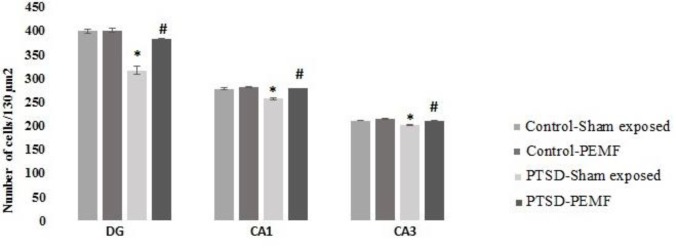
Number of cells in hippocampal CA1, CA3, and Dentate gyrus regions, per130 µm^2^ in four experimental groups

* *P*<0.001 as compared with PTSD-Sham exposed group

# *P*<0.001 as compared with Control-Sham exposed group

**Figure 8:**
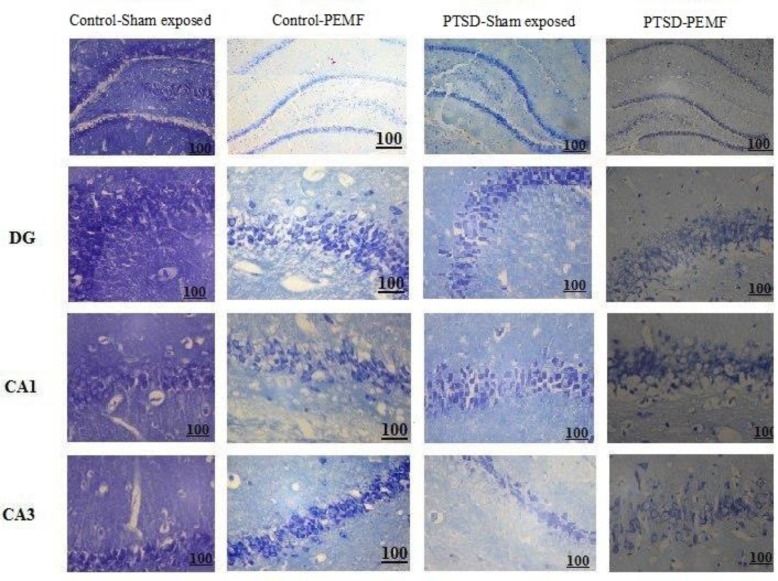
The photomicrographs of hippocampal CA1, CA3, and Dentate gyrus regions in four experimental groups

**Figure 9:**
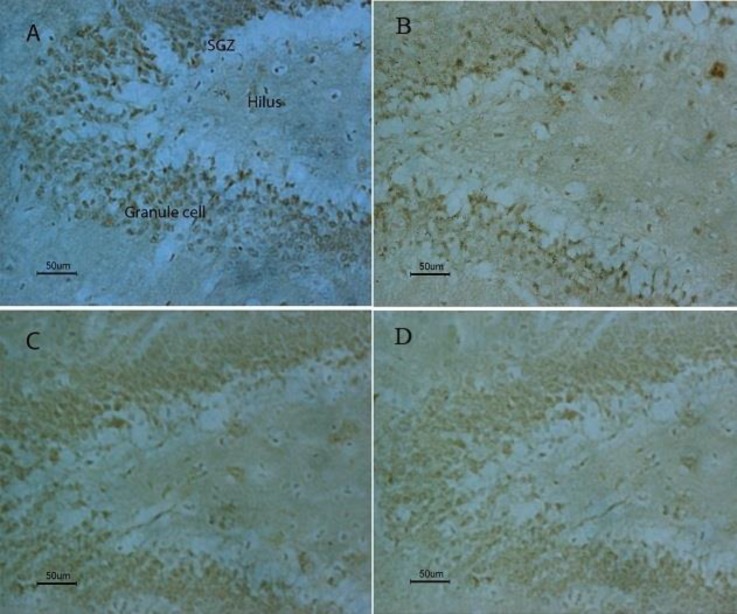
The results of BrdU immunohistochemistry in the hippocampus; cell proliferation results. A: PTSD- PEMF group, B: PTSD-Sham exposed group, C: Control-Sham exposed group and D: PEMF group

***Experimental design*** ***and animal grouping: ***

The rats were randomly divided into four groups Control-Sham exposed (n = 25), Control-PEMF (n = 25), PTSD-Sham exposed (n = 20), and PTSD-PEMF (n = 20). As illustrated in Figure 1, animals of the control groups, were conditioned on the contextual fear conditioning apparatus, then subjected to daily RMS (Control-PEMF group) or Sham RMS procedure (Control-Sham exposed group) for 14 days, three behavioral tests in three continuous days: open field test, elevated plus maze (EPM), and finally histological experiments. The PTSD groups were subjected to the single prolonged stress (SPS) procedure, as PTSD induction method, conditioned a day later, then, similar to the control groups, they passed the tests.

***Contextual fear conditioning apparatus***

An automated rodent fear conditioning system (TSE, Bad Homburg, Germany) was used to study contextual fear conditioning of each rat. Contextual fear conditioning took place in a conditioning box (45 cm×45 cm×47 cm). The walls and the ceiling of the box were constructed with clear Plexiglass, containing a loudspeaker and a light bulb providing dim illumination. The floor of the box was made of 28 stainless steel rods (6 mm in diameter, 12 mm gapped), where foot shock could be delivered from a constant current source. The box was enclosed in a sound attenuating chamber. The chamber was illuminated using a single house light and was cleaned by water before and after utilization. A software program (Etho Vision, version 3) was used to control the test in the box, and to collect, display, and store all experimental data for off-line analysis (17).

***Single prolonged stress procedure***

SPS is an animal model of PTSD that was first proposed by Liberzon *et al.* (1997, 1999) (18, 19). Briefly, rats were restrained for 2 hr, immediately followed by forced swimming for 20 min in 24 ^°^C water contained in a clear acrylic cylinder (24 cm in diameter and 50 cm in height). After 15 min of recuperation, animals were exposed to diethyl ether for 1–2 min until they lost consciousness (around 7–10 min). 

***Shock application and test of conditioned fear response***

One day after SPS, the stressed rats received electrical foot shock within the shock chamber: 180 sec after placement 4 sec, 1 mA shock administered via the metal grid. The stressed rats were held in the shock chamber for another 60 sec before they were returned to the home cages. To assess the conditioned fear response, a week after shock application, over three continuous days, rats were placed back in the chamber for 15 min periods without further shock application; and duration of freezing (absence of all visible movement except for respiration) was evaluated.

***Sensitized fear response test***

To perform the sensitized fear test, we used the open field apparatus. In this experiment, each rat was placed at the center of a cubic chamber (100× 100 × 60 cm). The test room was dimly illuminated with indirect light. The floor of the arena was divided into 16 equal squares (4×4cm). Center denoted the number of entries into the four central squares divided by the number of entries into the total squares. Behavior was analyzed by using the Ethovision ver 5 software package. The testing sessions lasted for 5 min, and the next test was performed after cleaning the chamber (17).

***Elevated plus maze***

EPM was made of wood consisting of two opposite-facing open arms (50 cm×10 cm), two opposite-facing closed arms (50 cm×10 cm×40 cm), and a central area measuring 10 cm×10 cm. The plus-maze was mounted on a base, raising it 50 cm above the floor. The animal was placed at the central area and faced the open arms. The count of a mouse’s entering/climbing onto the open/closed arms and the time mice spent on each arm were recorded in a 5 min test session using a video camera. An entry was defined as placing four paws onto the open arm. Four measures of behavior in the plus maze were scored: (1) time spent in the open arms; (2) time spent in the closed arms; (3) the number of entries into the open arms; (4) the number of entries into the closed arms. Two main factors to compare across the groups were: OAE = numbers of entries into the open arms/ (numbers of entries into the open arms + closed arms) and OAT = time spent in the open arms/ (time spent in the open arms + closed arms) (20).

***PEMF generation***

PEMF was applied using an electromagnet generator device (Model Magno 915X, Novin Medical Engineering Company, Iran) (Figure 2A). The exposure unit comprised a solenoid coil with an inner 290 mm diameter and 3.2 ohm resistance (Figure 2B). The frequency and intensity of the magnetic field were tuned by the regulator attached to the solenoid (Figure 2C). PEMF was applied continuously with an intensity of 7 mT, 30 Hz (16 min daily, 14 days) (21). Generator output frequency was confirmed by an oscilloscope (GOS-622B, Good Will Instruments Co. Malaysia), and magnetic field intensity was verified in the center of the coil with a Teslameter (CT3-A, Yuxiang Co, China). To reduce stress, animals were gradually acclimated for 10 min/day to a plastic cube and habituated to the noise induced by the stimulator for 1 week. Rats were placed individually in a cubic plastic container, covered by a plastic cap, and the container was fully fitted into the solenoid. Animals received whole-body exposure. Magnetic field’s exposure parameters were the same and unchanged for all animals. No signs of seizure or abnormal behavior were noticed in groups throughout the experiment (21). 

***Treatment protocol***

The Control-Sham exposed group was placed in the fear conditioning chamber and after 3 min received a 1 mA shock administered via the metal grid. Stressed rats were held in the shock chamber for another 60 sec before being returned to the home cages. They also received sham PEMF treatment (magnetic field off) on a daily basis (16 min/14 days). Control-PEMF animals, same as the Sham exposed group were conditioned in the shock chamber, but they received PEMF treatment (magnetic field on). PTSD-Sham exposed rats were conducted in the procedures described in the SPS section and then experienced all procedures as PEMF animals (6). PTSD-PEMF rats were conducted in the procedures described in the SPS section and then underwent all procedures as the PEMF group. The timeline of the experiments was illustrated in Figure 3. 

***Histological methods***

*Nissl staining*

Nissl stained sections were analyzed to determine the density of the cells in different layers of the hippocampus. Immediately after the behavioral tests rats were anesthetized IP with a mixture of ketamine (100 mg/kg, Sigma-Aldrich, USA) and xylazine (4 mg/kg) and were perfused intracardially with 0.1 M phosphate buffer for 10 min followed by phosphate-buffered paraformaldehyde 4% for 15 min (22).

*Infiltration and embedding*

The brains were removed and dehydrated through a graded series of alcohols (50%, 60%, 70%, 80% for 1 hr each, 90% and 96% for 1.5 hr each, and twice 100% for 1.5 hr) before infiltration. After dehydration, clearing, and impregnation, the hippocampal blocks were embedded in disposable tissue molds (22). 

*Staining*

Four coronal sections (10 μm) from each animal brain were cut at the level of the dorsal hippocampus and stained using cresyl violet (n=8, in each group). The staining solution contained 0.5 g cresyl violet dissolved in 100 ml distilled water. The mounted sections were placed in the staining solution for 20–30 min at room temperature and differentiated in 0.25% acetic acid until most of the stain was removed (4–8 sec). Then they were shortly passed through pure alcohol into xylene and were checked microscopically. If necessary, the differentiation would be repeated. Then the sections were cleared with xylene, and the covers were lipped, using Entellan. Number of pyramidal cells in a 130 μm^2^ segment of each of the hippocampal CA1 and CA3 stimulations and granule cells in the dentate gyrus was counted using light microscopy at 100× magnifications (22) 

*Administration of bromodeoxyuridine (BrdU)*

 Each group was divided into two subgroups; proliferation or survival tags were added to the group names (n= 8 in each) to determine the proliferation and survival of neurons. Throughout the manuscript, the terms proliferation group and survival group are used primarily to indicate that these animals were submitted to a different BrdU-labeling schedule. To evaluate the effects of various treatments on the survival of the newborn cells, animals of the survival group were injected twice with 5-bromo-2’-deoxyuridine (2×200 mg/kg BrdU [Sigma-Aldrich,USA] in saline, IP) on 2 consecutive days before the RMS treatment procedures started and perfused at the end of the experiment. To assess the effect of RMS treatment on the proliferation rate of cells, animals of the proliferation group received a single injection of BrdU (200 mg/kg IP), 24 hr before perfusion. The experimental procedure is illustrated in Figure 3.

*BrdU immunohistochemistry*

Animals were deeply anesthetized with a Ketamine/Xylazine mixture (xylazine 4 mg/kg, ketamine 100 mg/kg) and perfused transcardially (23). Brain fixation was done through transcardial perfusion with a fixative solution containing 4% paraformaldehyde in 0.1 M phosphate buffer pH 7.3. After the perfusion, the brains were removed and stored overnight in a fixative solution that is used for perfusion and then were processed for paraffin embedding and were sectioned into 5 µm thicknesses. Sections were taken from the hippocampus. A series of sections was used for BrdU immunostaining (24). 

The sections were deparaffinized and rehydrated. Then they were retrieved in a microwave by sodium citrate buffer for 10 min at 95–100 ^°^C and kept for 10 min at room temperature. The slides were washed twice, each time for 5 min in TBS plus 0.025% Triton X-100. Afterward, sections were incubated in blocking serum containing 10% Triton for 1 hr at room temperature. For BrdU, slides were incubated with mouse monoclonal anti-BrdU antibody (Sigma, Aldrich-USA) at 4 ^°^C, overnight. Then, they were washed twice with PBS and labeled with secondary antibody HRP for 1 hr at 37 ^°^C. Finally, all slides were washed twice, for 5 min each time, in PBS and the tissue slides were incubated with DAB for 5 min at room temperature. Then, the sections were gently rinsed with distilled water. After 20 min, all slides were incubated with 70%, 80%, and 100% ethanol, each for 5 sec, and xylene for 20 min. Finally, coverslips were mounted on slides using a mounting solution. Photos of slides were taken using a digital camera (Nikon, DXM 120, USA).

***Data analysis***

The results were expressed as mean ± SEM. Data were analyzed with two-way analysis of variance (ANOVA). Tukey’s *post hoc* test was performed to determine the source of detected significant differences. Values of *P*<0.05 were considered significant.

## Results

***Conditioned fear extinction test***

Figure 4 shows freezing responses of different groups as assessed by the time spent freezing during the 15 min test. Three tests over three consecutive days were performed.

Test 1:

A two-way ANOVA on freezing response data revealed no significant effect of groups (F _1, 86_= 0.556; *P*=0.458), a significant effect of PEMF treatment (F _1, 86_ = 12.555; *P*=0.001) and a significant interaction (F _1, 86_ =17.362; *P*=0.000). *Post hoc* comparison indicated that the freezing response of PTSD-PEMF group was significantly lower (*P*< 0.001) than those of PTSD-Sham exposed. Moreover, the freezing response of PTSD-Sham exposed group was significantly higher than Control-Sham exposed group (*P*<0.001). There was no significant difference between freezing behaviors of Control-PEMF and Control-Sham exposed groups (*P*>0.05). 

Test 2: 

A two-way ANOVA on freezing response data for the second day revealed a significant group effect (F _1, 86_ = 45.351; *P*=0.000), PEMF treatment (F _1, 86_ = 12.503; *P*=0.001), and Group × PEMF (F _1, 86_ =16.525; *P*=0.000). *Post hoc* comparison, as Test 1, indicated that freezing response of the PTSD-PEMF group was significantly lower (*P*<0.001) than those of the PTSD-Sham exposed. Moreover, the freezing response of PTSD-Sham exposed group was significantly higher than that of the Control-Sham exposed group (*P*<0.001). There was no significant difference between freezing behaviors of Control-PEMF and Control-Sham exposed groups and also between PTSD-PEMF and Control-PEMF groups (*P*>0.05).

Test 3:

Analyzing the Test 3 data revealed significant effect of groups (F _1, 86_ = 21.168; *P*=0.000), a PEMF treatment (F _1, 86_ = 6.823; *P*=0.011), and interaction (F _1, 86_ =10.205; *P*=0.002). *Post hoc* comparison indicated that the freezing response of PTSD-PEMF group was significantly lower (*P*<0.001) than those of PTSD-Sham exposed. Moreover, the freezing response of PTSD-Sham exposed group was significantly higher than Control-Sham exposed group (*P*<0.001). There were no significant difference between freezing behaviors of Control-PEMF and Control-Sham exposed groups and also between PTSD-PEMF and Control-PEMF groups (*P*>0.05). 

These findings indicate that the PTSD procedure resulted in an increased freezing response as compared to the control group. Moreover, PEMF treatment reduced this response.

***Sensitized fear test***

Two indices, total crossing and center square’s crossing of different groups in the open field test (OFT) were measured. Less sensitized animals will cross more, especially from center squares of the open field. Two-way ANOVA of the center square’s crossing results showed significant effect of groups (F 1, 86 = 29.35; *P*=0.000), PEMF treatment (F 1, 86 = 29.012; *P*=0.000), and interaction (F 1, 86 =22.657; *P*=0.000). *Post hoc* comparison indicated that in the PTSD-PEMF group (3.5 ± 0.46), center crossing increased significantly compared with the PTSD-Sham exposed group (0.3 ± 0.17, *P*<0.001). There was no significant difference between Control-Sham exposed (0.625 ± 0.14) and Control-PEMF (0.791 ± 0.21) groups (Figure 5A). 

Two-way ANOVA of the total crossing results showed no significant effect of groups (F 1, 86 = 0.203; *P*=0.653), PEMF treatment (F 1, 86 = 35.461; *P*=0.000), and interaction of Group × PEMF (F 1, 86 =77.026; *P*=0.000). *Post hoc* comparison indicated that in the PTSD-PEMF group (81.75 ± 4.9), total crossing increased significantly as compared with the PTSD-Sham exposed (27.8 ± 1.7, *P*<0.001) and Control-PEMF (47.91 ± 2.9, *P*<0.001) groups. There was no significant difference between Control-Sham exposed (58.28 ± 4.0) and Control-PEMF (47.91 ± 2.9) groups (Figure 5B).

***Anxiety-like behavior***

The percentage of time spent in the open arms and also the number of entries into the open arms of the EPM were evaluated (Figure 6). Analysis of OAT data revealed significant effects of groups (F _1, 86_ =51.128; *P*=0.000), RMS treatment (F _1, 86_ =192.43; *P*=0.000), and interaction of groups and RMS treatment (F _1, 86_ =122.48; *P*=0.000).

The number of entries into the open arms as a percentage of total number of arm entries OAE was also evaluated. Significant interaction (F _1, 86_ =9.398; *P*=0.003), effect of treatment (F _1, 86_ =9.346; *P*=0.003), and no significant effects of groups (F _1, 86_ =0.343; *P*=0.559) were observed.

OAT and OAE in the PTSD-PEMF group were increased as compared with the PTSD-Sham exposed, Control-Sham exposed, and Control-PEMF groups (Figure 6). The PTSD-Sham exposed group and the Control-Sham exposed group had a significant difference in OAT and OAE (*P*<0.01). Less anxious animals will spend more time in the open arms of the elevated plus maze.

***Histological results***

*Number of CA1 pyramidal cells*

Two-way ANOVA showed significant effect of groups (F _1, 28_ =47.822; *P*=0.000), RMS treatment (F _1, 28_ =54.746; *P*=0.000), and interaction of group × PEMF (F _1, 28_ =22.71; *P*=0.000). *Post hoc* comparisons indicated that the number of CA1 pyramidal cells in the PTSD-PEMF and Control-Sham exposed groups were significantly more than PTSD-Sham exposed group (*P*<0.001). There was no significant difference between Control-Sham exposed and Control-PEMF groups (*P*>0.05) (Figures 7 and 8).

*CA3 pyramidal cells*

Two-way ANOVA showed significant effect of groups (F _1, 28_ =90.28; *P*=0.000), RMS treatment (F _1, 28_ =90.2; *P*=0.000), and interaction of group × PEMF (F _1, 28_=19.21; *P*=0.000). *Post hoc* comparisons indicated that the number of CA1 pyramidal cells in the PTSD-PEMF and Control-Sham exposed groups was significantly more than PTSD-Sham exposed group (Figures 7 and 8).

*DG neurons*

The total number of DG neurons was significantly different among groups (F _1, 28_ =76.619; *P*=0.000), PEMF treatment (F _1, 28_ =33.704; *P*=0.000), and interaction of group × PEMF (F _1, 28_ =31.038; *P*=0.000). *Post hoc* comparisons indicated that the number of DG granule cells in the PTSD-PEMF and Control-Sham exposed groups were significantly more than PTSD-Sham exposed group (*P*<0.001). There was no significant difference between Control-Sham exposed and PEMF groups (*P*>0.05) (Figures 7 and 8). 

***Results of BrdU immunohistochemistry***

*Cell proliferation results*

Cell proliferation was addressed by BrdU labeling to assess the effect of PTSD and/or PEMF treatment. Gross examination revealed decreased immunostaining for BrdU in DG and hilus of PTSD-Sham exposed, as compared with Control-Sham exposed, although cell numbers were not quantified (Figure 9). There were more BrdU-positive cells in the dentate gyrus of the PTSD-PEMF group as compared with the PTSD-Sham exposed group. The majority of BrdU-positive cells were the same as that in Control-Sham exposed and Control-PEMF groups. Treatment with PEMF alone without SPS did not affect cell proliferation. 

*Cell survival results*

To determine the effect of PTSD and/or PEMF treatment on the survival of cells, animals were injected with BrdU, twice, after fear conditioning and before PEMF procedure. Qualitative observations showed that the number of surviving BrdU-positive cells in PTSD-Sham exposed group was lower than those of Control-Sham exposed and also Control-PEMF group. RMS resulted in an increased number of BrdU-labeled cells in the PTSD-PEMF group as compared with PTSD-Sham exposed group. Treatment with PEMF alone did not reach a significant difference between the two control groups. The relevant data is not shown. 

## Discussion

This study aimed to determine whether PEMF, after fear conditioning, facilitates fear extinction and alleviates depression and stress-related responses in the PTSD animal model. Also, it was to inspect whether the mentioned effects of PEMF were caused by the effects on hippocampal neurogenesis in PTSD rats.

This report is the first to assert that PEMF facilitates the extinction of fear memory in PTSD rats. We assessed the effects of 7 mT, 30 Hz electromagnetic field after SPS induction. One day after the model was established, the rats were conditioned and then treated with PEMF for two weeks. Fear responses of the rats were estimated using the level of freezing upon context stimulus and were compared between the PEMF and corresponding sham groups. The control rats treated with PEMF showed no difference in freezing percentage when compared with the Control-Sham exposed group. However, the PTSD rats treated with PEMF showed significantly less freezing behavior during extinction tests than the PTSD-Sham exposed group, and this enhancement of fear extinction remained after 48 hr (on Test 3), without further stimulation. This finding suggests that PEMF following trauma enhances fear extinction and that PEMF, in combination with exposure therapy is potentially useful for facilitating extinction memory in the treatment of PTSD. Co-treatment with a magnetic field and exposure therapy are also useful for the treatment of anxiety and sensitized fear in PTSD.

In agreement with our study, some previous studies have suggested EMF may interact with learning and memory processes (25, 26). Transcranial magnetic stimulation has been suggested as a potential candidate for the treatment of dysregulated fear memory and also stimulation of the infralimbic region was found facilitating fear extinction in rats (27). In line with our results, a previous study indicated that high-frequency rTMS paired with trauma reminders enhances fear extinction (28). 

PEMFs interact with different brain nuclei and neurotransmitter systems in different ways; they do not affect all parts of the brain fixedly. Since any behavior is performed under the control of a given nucleus, and each nucleus is not affected by the RMS at the same time, RMS exposures with specific parameters may affect different behaviors (26, 29). 

What is the possible neurophysiological mechanism of facilitated fear extinction in PTSD rats, as induced by PEMF? We have probed the possibility that maybe this increase in extinction is due to the effects of PEMF on the brain area that is structurally related to the incidence of memory extinction. We speculate that this mechanism may be the modulation of cell proliferation and survival rates in the area involved in fear extinction, possibly the hippocampus. Cell proliferation and survival were addressed by BrdU labeling to recognize the effect of 14 days of PEMF treatment. In the present study, the proliferation and survival rates of PTSD rats were normalized by PEMF. There were no differences between the proliferation and survival rates of cells in the two control groups; it means that PEMF doesn’t affect the proliferation and survival rates in conditioned, healthy rats.

Our results are in line with the above studies that showed rTMS increased adult hippocampal neurogenesis in rats (12), and was effective in clinical treatments with a magnetic field (30, 31). 

Neuroprotective-like effects of magnetic fields has been reported by previous studies that suggested that the neuroprotection is due to various reasons, maybe by preventing the creation of the necessary conditions for oxidative stress and apoptosis, by stimulating the release of neurotrophic factors, neurotransmitter release excitation, affecting the glial system, and so on (32, 33). 

SPS and PTSD-induced suppression of cell proliferation is associated with neuronal atrophy and cell loss in the hippocampal dentate gyrus. Neurotrophins have a crucial role in brain development, survival, and maintenance of neuronal functions, and synaptic plasticity. BDNF with other growth factors promotes the survival and neurogenesis of neural cells including hippocampal neurons (33-35). Besides, some studies have shown that a magnetic field increases serum BDNF levels in animals (36-38). We did not measure the BDNF levels, but this is probably one of the possible mechanisms of PEMF effects on the cell density.

Extremely low-frequency magnetic stimulation induced a reduction in cell damage biomarkers, oxidative stress (lipid peroxidation products, protein carbonyls, GSH, and catalase) and reversed the neurodegenerative process. It provides a protective effect against oxidative damage either by affecting the mitochondrial activity or by the expression of proteins transcription (39).

Oxidative stress may be a factor in neuronal loss in PTSD, and reactive oxygen species are involved in its pathogenesis. Transcranial magnetic stimulation can partially modulate the imbalance in the oxidative stress system in favor of tissue protection (40).

In a study, it was indicated that rTMS significantly enhanced expression of Bcl-2 and reduced expression of BAX, which are known as the anti-apoptotic and pro-apoptotic proteins, respectively (41). Therefore, the protective effect of PEMF may be due to its anti-apoptotic property. Further research is needed to determine the mechanism of PEMF neuroprotection in PTSD patients.

PTSD can produce more robust symptoms and enhance conditioned and sensitized fear responses (42). In line with previous studies, PTSD-induction enhanced sensitized fear response in PTSD-sham exposed group as compared with control-sham exposed group. 

PEMF attenuates PTSD-induced exaggerated sensitized fear, it significantly reduced sensitization, as PTSD-PEMF group had a significantly larger total and latency center squares crossing in open field test than that observed from the PTSD-Sham exposed group.

This effect is not related to the PEMF effect on decreasing locomotor activity, because there was no significant difference between Control-Sham exposed and PEMF groups. Magnetic field had a specific effect on improving the PTSD-induced sensitized fear responses. The reduction of fear by magnetic field exposure observed in the present study is in agreement with similar results obtained with rats exposed to rTMS, through a different procedure (43). Although magnetic resonance associated stimulations also increase self-grooming (44), we did not observe any difference in grooming between groups.

Enhancement of anxiety in the PTSD-sham exposed group can be realized by reducing the percentage of entry to the open arm of the elevated plus maze, as compared with Control-Sham exposed group. In the present study, we showed the anxiolytic effects of a magnetic field in PTSD animals. The effects of magnetic field on anxiety are highly dependent on the properties and duration of the field, the type of anxiety induction model, and even the strain of experimental animal. Therefore, the present study, in line with many studies, confirms the anxiolytic effects of magnetic stimulation (45-47) but contradicts the studies that have reported rTMS is anti-depressant but not anxiolytic in rats (48).

## Conclusion

The present study demonstrated an attenuation of PTSD-induced failure of conditioned fear extinction and exaggerated sensitized fear in rats using 14-day PEMF, and it appeared that the increase might be related to the neuroprotective effects of the magnetic field on the hippocampus. The results of this study showed that PEMF could increase the neurogenesis in the hippocampus of the PTSD rats and that, on the other hand, the behavioral disorders in PTSD rats treated with PEMF also improved. It is possible that PEMF has corrected behavioral disorders in the PTSD rats by its positive effects on neurogenesis. Further studies are needed to examine the mechanism more precisely‏.

Also, we observed an anxiolytic effect of magnetic fields in PTSD rats. These data indicate the protective effect of PEMF and its possible benefits for therapeutic strategies.

In addition, we observed an anxiolytic effect of magnetic fields in PTSD rats. These data indicate the protective effect of PEMF and its possible application in therapeutic strategies.

## Conflicts of Interest

The authors declare that they have no conflicts of interests.
